# Motor cortex and pain control: exploring the descending relay analgesic pathways and spinal nociceptive neurons in healthy conscious rats

**DOI:** 10.1186/s12993-019-0156-0

**Published:** 2019-03-25

**Authors:** Patrícia Sanae Souza Lopes, Ana Carolina Pinheiro Campos, Erich Talamoni Fonoff, Luiz Roberto Giorgetti Britto, Rosana Lima Pagano

**Affiliations:** 1 Laboratory of Neuroscience, Hospital Sírio Libanês, São Paulo, SP 01308-060 Brazil; 2 Department of Physiology and Biophysics, Institute of Biomedical Sciences, University of São Paulo, São Paulo, SP 05508-900 Brazil; 3 Department of Neurology, School of Medicine, University of São Paulo, São Paulo, SP 01060-970 Brazil

**Keywords:** Motor cortex, Neurostimulation, Antinociception, Raphe nuclei, Spinal cord

## Abstract

Motor cortex stimulation (MCS) is an effective therapy for refractory neuropathic pain. MCS increases the nociceptive threshold in healthy rats via endogenous opioids, inhibiting thalamic nuclei and activating the periaqueductal gray. It remains unclear how the motor cortex induces top-down modulation of pain in the absence of persistent pain. Here, we investigated the main nuclei involved in the descending analgesic pathways and the spinal nociceptive neurons in rats that underwent one session of MCS and were evaluated with the paw pressure nociceptive test. The pattern of neuronal activation in the dorsal raphe nucleus (DRN), nucleus raphe magnus (NRM), locus coeruleus (LC), and dorsal horn of the spinal cord (DHSC) was assessed by immunoreactivity (IR) for Egr-1 (a marker of activated neuronal nuclei). IR for serotonin (5HT) in the DRN and NRM, tyrosine hydroxylase (TH) in the LC, and substance P (SP) and enkephalin (ENK) in the DHSC was also evaluated. MCS increased the nociceptive threshold of the animals; this increase was accompanied by activation of the NRM, while DRN activation was unchanged. However, cortical stimulation induced an increase in 5HT-IR in both serotonergic nuclei. MCS did not change the activation pattern or TH-IR in the LC, and it inhibited neuronal activation in the DHSC without altering SP or ENK-IR. Taken together, our results suggest that MCS induces the activation of serotonergic nuclei as well as the inhibition of spinal neurons, and such effects may contribute to the elevation of the nociceptive threshold in healthy rats. These results allow a better understanding of the circuitry involved in the antinociceptive top-down effect induced by MCS under basal conditions, reinforcing the role of primary motor cortex in pain control.

**Electronic supplementary material:**

The online version of this article (10.1186/s12993-019-0156-0) contains supplementary material, which is available to authorized users.

## Introduction

Epidural motor cortex stimulation (MCS) is an effective therapeutic option for refractory neuropathic pain of either peripheral or central origin [59, 60, 62, 74, 93, 97]. MCS provides pain relief in 55 to 64% of patients refractory to other treatments [13, 15, 42, 44, 88], but the neural mechanism underlying its analgesic effect remains poorly understood. In patients with neuropathic pain, MCS modulates nociceptive spinal reflexes [21] and induces analgesia by activating top-down controls originating from intracortical horizontal fibers or interneurons [39]. Electrophysiology and functional imaging studies have shown that MCS activates supraspinal areas involved in the perception and/or emotional appraisal of pain, including the lateral thalamus, anterior cingulate cortex (ACC), anterior insula and periaqueductal gray (PAG) [20, 21, 70, 71]. The analgesic effect of MCS is also correlated with the release of endogenous opioids in the ACC, insula and PAG [47, 48]. In healthy rats, with no neuropathic conditions, MCS reduces the responsiveness of spinal nociceptive neurons [16, 80], thereby raising the nociceptive threshold via endogenous opioids [12], this response is associated with inhibition of the thalamic nuclei and activation of the PAG [65]. In neuropathic rats, MCS reverses central and peripheral pain [8, 45, 64, 78, 96, 99, 100], activating the limbic system and PAG and inhibiting the thalamic nuclei and spinal nociceptive neurons [64]. It has been hypothesized that, in humans and animals, MCS induces analgesia by activating the descending analgesic pathways [12, 70, 71, 100]; however, it is not clear which midbrain nuclei are modulated after cortical stimulation or how they act on spinal nociceptive neurons to elevate the nociceptive threshold.

The serotonergic dorsal raphe nucleus (DRN) is an important brainstem nucleus involved in the pain modulation system, which target different brain areas because its widespread ascending and descending projections, including a few fibers to the spinal cord directly [101]. Other pivotal areas involved in the descending analgesic system include the opioidergic PAG, the noradrenergic locus coeruleus (LC), and the rostral ventromedial medulla (RVM), which consists of the serotonergic nucleus raphe magnus (NRM) and adjacent nucleus reticularis magnocellularis [4, 10, 55]. Activation of these descending pathways induces the release of serotonin (5HT) and noradrenaline (NA) in the dorsal horn of the spinal cord (DHSC); these neurotransmitters acting on 5HT_1A_ and α2-adrenergic receptors directly or indirectly inhibit projection neurons, central terminals of primary afferent fibers and excitatory interneurons by decreasing the release of excitatory neurotransmitters, such as glutamate, substance P (SP) and calcitonin gene-related peptide (CGRP) [10, 106]. A dysregulation in descending pain modulation is observed in persistent pain condition, since that central sensitization phenomena changes the subtype of spinal 5HT/NA receptors, which can have inhibitory or facilitatory role on pain [43, 69, 85, 102]. Additionally, the descending analgesic system induces the activation of inhibitory interneurons in the DHSC; these neurons release GABA, glycine and enkephalin (ENK), which also contribute to the inhibition of ascending nociceptive transmission [4, 106].

Considering pain control induced by cortical stimulation, while descending noradrenergic pathway may not be critical for the MCS-induced analgesia in healthy or neuropathic rats [99], descending serotonergic and dopaminergic pathways contribute to spinal antinociception induced by MCS in neuropathic rats [98, 100]. However, its role in spinal modulation is not totally clear. Therefore, a more thorough investigation of the role of motor cortex in the nociceptive threshold in healthy conscious rats could facilitate the understanding of its function in pain control. Considering that the neurocircuitry involved in MCS-induced analgesia needs to be better clarified, we investigated the activation pattern of the serotonergic and noradrenergic nuclei involved in the descending analgesic system, as well as their effect on the spinal nociceptive neurons, in response to MCS in healthy rats.

## Materials and methods

### Experimental design

**Fig. 1:**
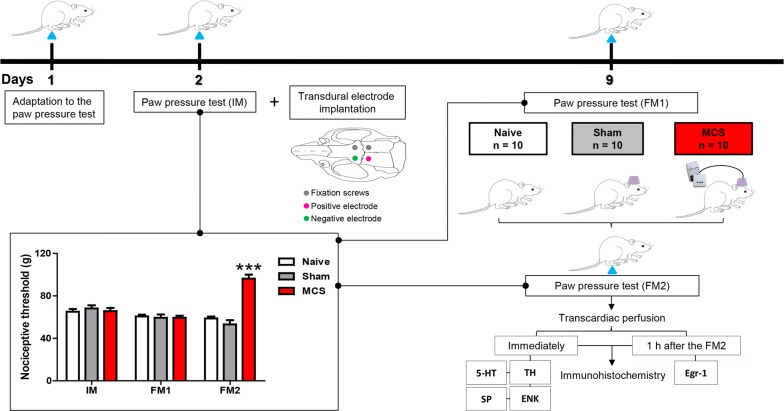
Experimental design of the study and effect of cortical stimulation on mechanical nociceptive threshold. Rats were habituated to the nociceptive test the day preceding the electrode implantation (day 1). The paw pressure test was conducted prior to the electrode implantation (initial measurement, IM; day 2) and 7 days later (day 9), as well as before (final measurement 1, FM1) and during MCS (final measurement 2, FM2). Naive and sham rats were also evaluated. Animals were divided into three groups: rats with no surgical procedure (Naive), rats with transdural electrodes and false stimulation (Sham), and stimulated rats (MCS). The transdural electrodes were implanted over the motor cortex of the left hemisphere, and the nociceptive threshold of the right hind paw was determined. Values represent the mean ± SEM of 10 animals from each group. Statistically significant differences from the naive group (***p < 0.0001, after Bonferroni’s *post hoc* test) are indicated. Half of the animals in each group were perfused immediately after the last nociceptive test and evaluated for 5HT-, TH-, SP- and ENK-IR; the other half was perfused 1 h after the last nociceptive test for evaluation of Egr-1-IR. *5HT* serotonin, *ENK* enkephalin, *IR* immunoreactivity, *MCS* motor cortex stimulation, *SP* substance P, *TH* tyrosine hydroxylase

Adult rats were evaluated in a nociceptive test (described in Measuring nociceptive threshold below), and subsequently, under anesthesia, transdural electrodes were implanted over the functional area of the left primary motor cortex associated with the right hind limb [12]. After 1 week, the nociceptive test was performed again, and a group of rats undertook one session of MCS (15 min); at the end of this period, while still under stimulation, they were re-evaluated on the test. Rats that underwent surgical procedures but were not electrically stimulated (sham) and rats that did not receive any surgical procedures (naive) were also evaluated. Immediately after the last nociceptive evaluation, the animals (5 animals per group) were anesthetized and immediately perfused to have their brains and spinal cords processed to evaluate immunoreactivity for 5HT, tyrosine hydroxylase (TH) and SP/ENK in the DRN/NRM, LC and DHSC, respectively. Additionally, another group of animals (5 animals per group) was anesthetized 1 h after the last nociceptive test and then subjected to an Egr-1 immunohistochemistry assay to evaluate the neuronal activation pattern in the DRN, NRM, LC and DHSC (Fig. 1).

### Animals

Thirty-six male Wistar rats (180–220 g) were housed in acrylic boxes (three rats per cage) for at least 5 days before the initiation of the experimental procedures. The boxes, containing wood shavings, were kept in a room with a stable, controlled ambient temperature (22 ± 2 °C) and a light/dark cycle of 12 h/12 h, and the animals had free access to water and rat chow pellets. All experimental procedures were in accordance with the guidelines for the ethical use of animals in research involving pain and nociception [108] and were reported in accordance with the ARRIVE guidelines (http://www.nc3rs.org.uk/arrive-guidelines). The study was approved by the Ethics Committees on the Use of Animals of both the Hospital Sírio Libanês (protocol number CEUA 2011/13) and the Institute of Biomedical Sciences of the University of São Paulo (no. 055, page 103, book 02, 2011).

### Electrode implantation and electrical stimulation parameters

Rats were deeply anesthetized with ketamine/xylazine (0.5/2.3 mg/kg, i.m.) and received a local scalp injection of 2% lidocaine (100 µL/animal, s.c.). Whenever necessary, supplementary doses of ketamine were administered to the animals to ensure an anesthetized state. Then, under stereotaxic guidance using a functional map developed by our group [14], two transdural stainless steel electrodes (cylinders of 0.8 mm in diameter) were fixed over the left primary motor cortex in the functional area corresponding to the right hind limb (1.0 mm rostral and 1.5 mm caudal to the bregma, 1.5 mm lateral to the midline). Two fixation screws (implanted 4 to 6 mm away from the site of stimulation) and acrylic polymer were used to stabilize the implant and to ensure electrical isolation. The contacts of each electrode pole were inserted into a connector, which was also fixed to the whole ensemble. For 3 consecutive days, the animals received anti-inflammatory (Ketoprofen 5 mg/kg/day, via s.c.) to prevent pain from the surgery. All implanted rats were allowed to recover for 1 week before testing began. Electrical stimulation was applied according to an earlier study, which showed changes in mechanical nociceptive threshold without interfering with thermal nociceptive threshold and with general or motor activities [12]. One week after implantation of the electrodes, electrical stimulation was delivered in a single 15-min session (1.0 V, 60 Hz, and 210 µs; Medtronic electrical stimulator, Minneapolis, MN), and the final measurements during the nociceptive test were recorded while the rats were still under stimulation. The cathode was always chosen to be the posterior contact of the electrode because, according to the functional map, that site has greater surface area corresponding to the hind limb [14]. The sham group was subjected to the same conditions but did not receive stimulation. The rats were randomly divided into the sham and stimulated groups.

### Measuring nociceptive threshold

On the day of the nociceptive tests, the animals were brought into a separate, quiet room 1 h before the tests to allow them to habituate to the environment. The mechanical nociceptive threshold was determined using a pressure apparatus (EEF-440, Insight, SP, Brazil), which has been previously described [73]. Briefly, a mass with increasing magnitude (16 g/s) was applied to the right hind paw. The mass (in grams) required to induce the withdrawal response represented the nociceptive threshold. Antinociception was defined as a significant increase in the pressure necessary to induce the withdrawal response in experimental animals compared either with initial measurement of the same animal and with the control animals (naive group). Aiming to reduce animal stress, the rats were handled by the experimenter and were habituated to the paw pressure test the day preceding the electrode implantation. Nociceptive tests were conducted prior to the electrode implantation (initial measurement) and 7 days later, as well as before (final measurement 1) and during MCS (final measurement 2). Naive and sham rats were also evaluated.

### Immunohistochemistry

The animals were divided into three groups: rats that had not undergone surgery (naive, n = 10), rats with electrodes implants, but not electrically stimulated (sham, n = 13), and stimulated rats (MCS, n = 13). Randomly, half of the animals in each group were perfused immediately after the last nociceptive test to evaluate 5HT, TH, SP and ENK immunoreactivity (IR), and the other half was perfused 1 h after the last nociceptive test to evaluate Egr-1-IR. In this last group, the animals were perfused 1 h after the last nociceptive stimulus because the expression levels of inducible transcription factor proteins (including c-Fos, c-Jun and Egr-1) peak at approximately 1 h after the stimulus and fade by 3 to 4 h [29]. Rats were deeply anesthetized with ketamine and xylazine and then subjected to transcardiac perfusion with saline solution followed by 4% paraformaldehyde (PFA) dissolved in 0.1 M phosphate buffer (PB). The brain and lumbar spinal cord (L4–L6 segments) were collected and postfixed in PFA for 4 h, followed by incubation with 30% sucrose solution in PB for 48 h at 4 °C. Tissue sections (30 µm) were cut on a freezing microtome, washed in PB, and incubated for 12 to 16 h at 4 °C with the following primary antibodies: rabbit anti-Egr-1 (Early growth response protein 1, C-19; Santa Cruz Biotechnology, CA, USA), rabbit anti-5TH (NT-102, Protos Biotech, NY, USA), mouse anti-TH (MAB5280, Millipore, MA, USA), rabbit anti-SP (AB962, Millipore) or mouse anti-ENK (MAB350, Millipore) diluted 1:1000 in 0.3% of Triton X-100 containing 5% normal donkey serum (Jackson ImmunoResearch, ME, USA). After being washed (3 × 10 min) with PB, tissue sections were incubated for 2 h at room temperature with biotinylated secondary antibodies (1:200, Jackson ImmunoResearch). After additional washes, the sections were incubated for 2 h at room temperature with avidin-biotin complex (1:100; ABC Elite kit, VectorLabs, CA, USA), and visualized with 0.05% diaminobenzidine tetrahydrochloride (DAB, Sigma-Aldrich, MO, USA) and 0.03% (final concentration) hydrogen peroxide in PB. The sections were then washed and mounted on glass slides, and the staining was intensified with 0.05% osmium tetroxide in water. Afterwards, the sections were dehydrated through graded ethanol solutions, followed by xylene, and coverslipped with Permount (Fisher Scientific, PA, USA). The IR was captured by means of a light microscope (Eclipse E1000, Nikon, NY, USA) and was analyzed using ImageJ software (National Institute of Health, MD, USA; http://rsbweb.nih.gov/ij/). Figures were assembled using Adobe Photoshop (Adobe Systems, CA, USA); the images were optimized using contrast and brightness only. Quantitative analysis was performed to determine the density of nuclei showing positive IR for Egr-1 in the dorsomedial and ventromedial DRN, NRM, LC and DHSC (laminae I–IV) in animals perfused 1 h after the behavior analyses. Additionally, the densities of 5HT-IR in the DRN and NRM, TH-IR in the LC and SP and ENK-IR in the DHSC were analyzed in the animals perfused immediately after the nociceptive tests. The areas analyzed were defined for each structure by using a 10× objective for the DRN (B7; − 7.32 to − 7.92 mm from bregma), NRM (B3; − 11.04 to − 11.76 from bregma) and LC (A6; − 9.72 to − 9.96 mm from bregma) [9] and a 40× objective for the DHSC (laminae I–IV). For each assay, total number of positive profile (immunostained particles) was used to provide a mean immunolabel value (compared with pre-defined threshold) in five tissue sections per animal and five animals per group. The regions of interest were identified according to histological landmarks based on the adjoining Nissl-stained sections in the brain atlas [67] and spinal cord atlas [56].

### Statistical analysis

Data are presented as the mean ± standard error of the mean (SEM). Statistical analyses were conducted with GraphPad Prism 5.0 software (GraphPad Software Inc; CA, USA). The results of the nociceptive tests were analyzed using two-way (2-w) repeated measures (rm) analysis of variance (ANOVA) followed by the Bonferroni’s post hoc test. Immunohistochemistry data were normalized (by defining the naive group as 100% for Egr-1 and 1.0 ratio for 5HT, TH, SP and ENK) and were analyzed using one-way (1-w) ANOVA followed by the Bonferroni’s post hoc test. In all cases, p < 0.05 was considered statistically significant.

## Results

Twenty-six animals underwent electrode implantation, as stated above; however, six implanted rats were excluded from the study because they removed their subdural implants before the final nociceptive tests. Hence, the results concern naive animals n = 10, sham animals n = 10 and MCS animals n = 10.

MCS increased the mechanical nociceptive threshold in rats in the paw contralateral to the stimulation side compared with the thresholds of the control group, non-operated naive animals (Fig. 1, Additional file 1: Table S1). The nociceptive threshold increased in 62% when compared to the nociceptive threshold observed in the naive animals (2-w-rm-ANOVA, Treatment × Time, F_2,42_ = 24.91, p = 0.0001; followed by Bonferroni’s *post hoc* test, p < 0.001; Fig. 1).

**Fig. 2:**
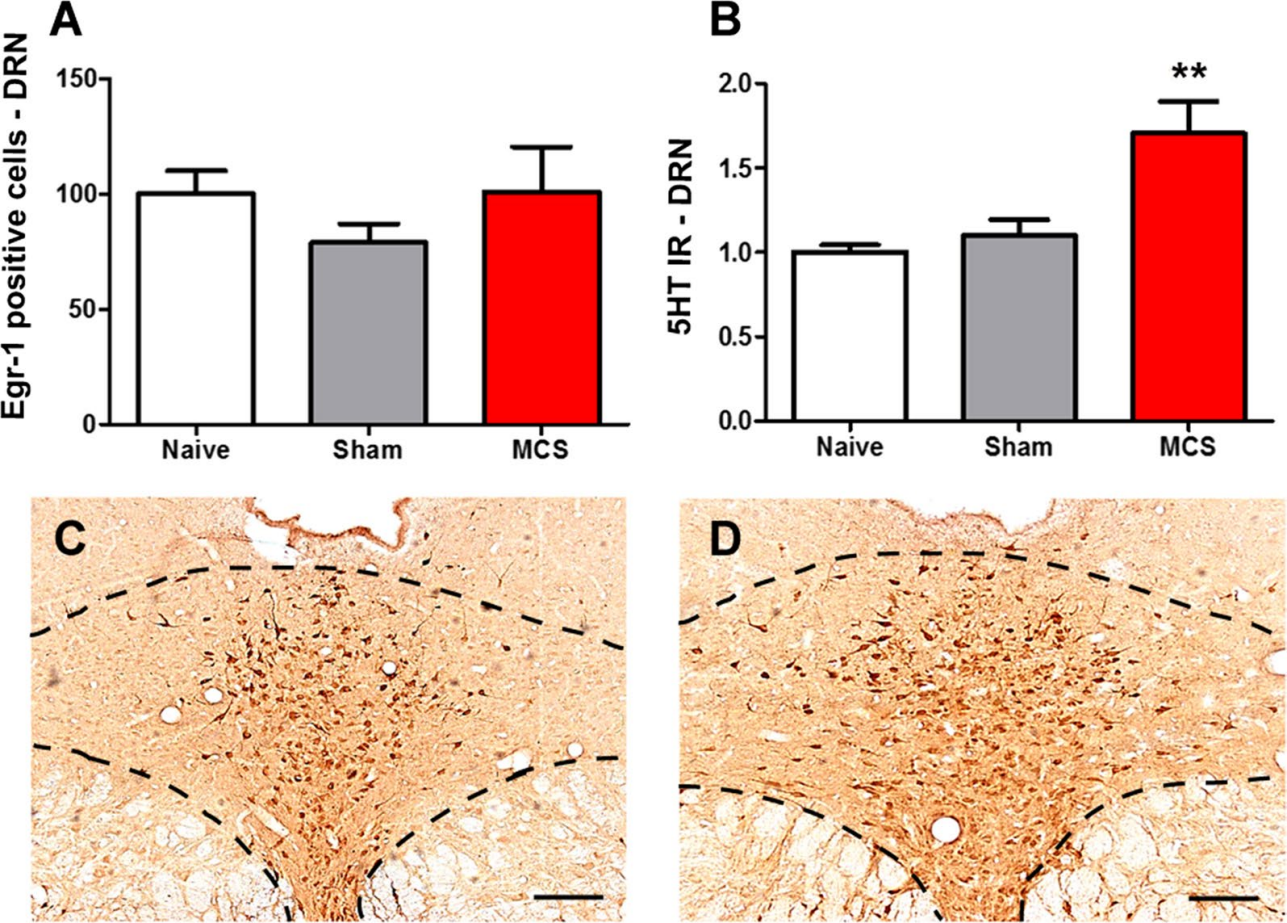
MCS, antinociception, and their correlation with NRM activation. Quantification of Egr-1 (**A**) and 5HT (**B**) IR in the NRM of naive (without surgical intervention), sham (with epidural electrodes but without stimulation) and stimulated (MCS, 1.0 V/60 Hz/210 µs, 15 min) rats. Values represent the mean ± SEM (n = 5 animals per group). **p < 0.01 compared to the naive group, after Bonferroni’s *post hoc* test. Photomicrographs illustrating the 5HT-IR in the NRM of sham (**C**) and stimulated (**D**) rats. *5HT* serotonin, *IR* immunoreactivity, *MCS* motor cortex stimulation, *NRM* nucleus raphe magnus. Scale bars: 200 μm

Regarding the NRM, we observed that naive and sham-stimulated animals presented the same pattern of neuronal activation and 5HT-IR in the NRM (Fig. 2A, B). MCS induced an increase of 70% in Egr-1-IR (1-w-ANOVA, F_(2,12)_ = 20.06, p = 0.0003, followed by Bonferroni’s *post hoc* test, p < 0.01; Fig. 2A) and 62% in 5HT-IR (1-w-ANOVA, F_(2,12)_ = 6.43, p = 0.0126, followed by Bonferroni’s *post hoc* test, p < 0.01; Fig. 2B–D) in the NRM over the levels in the naive group (Additional file 1: Table S1).

**Fig. 3:**
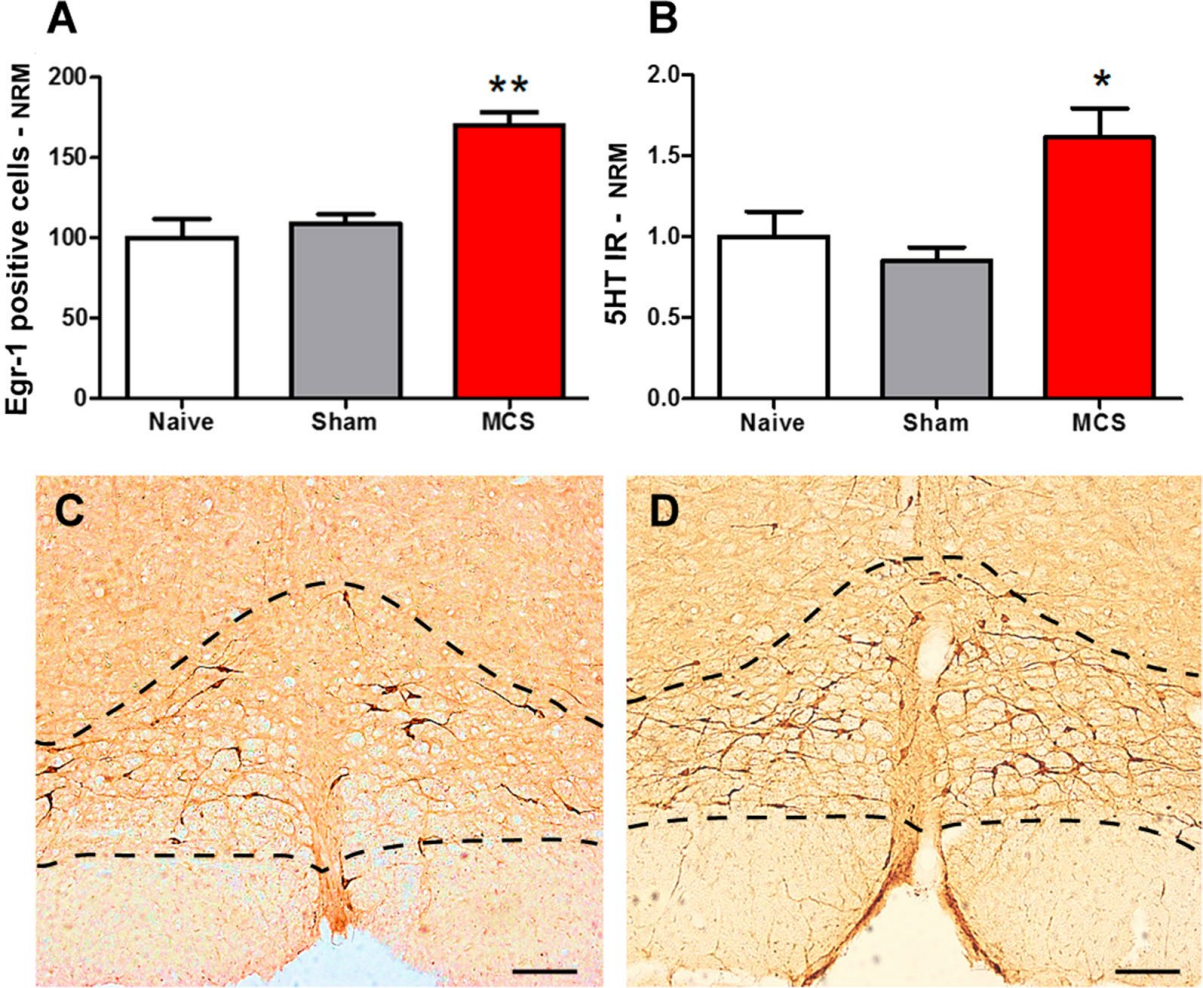
Participation of the DRN in MCS-induced antinociception. Quantification of Egr-1 (**A**) and 5HT (**B**) IR in the DRN of naive (without surgical intervention), sham (with epidural electrodes but without stimulation) and stimulated (MCS, 1.0 V/60 Hz/210 µs, 15 min) rats. Values represent the mean ± SEM (n = 5 animals per group). *p < 0.05 and **p < 0.01 in comparison to the naive group, after Bonferroni’s *post hoc* test. Photomicrographs illustrating the 5HT-IR in the DRN of sham (**C**) and stimulated (**D**) rats. *5HT* serotonin, *DRN* dorsal raphe nucleus, *IR* immunoreactivity, *MCS* motor cortex stimulation. Scale bars: 200 µm

**Fig. 4:**
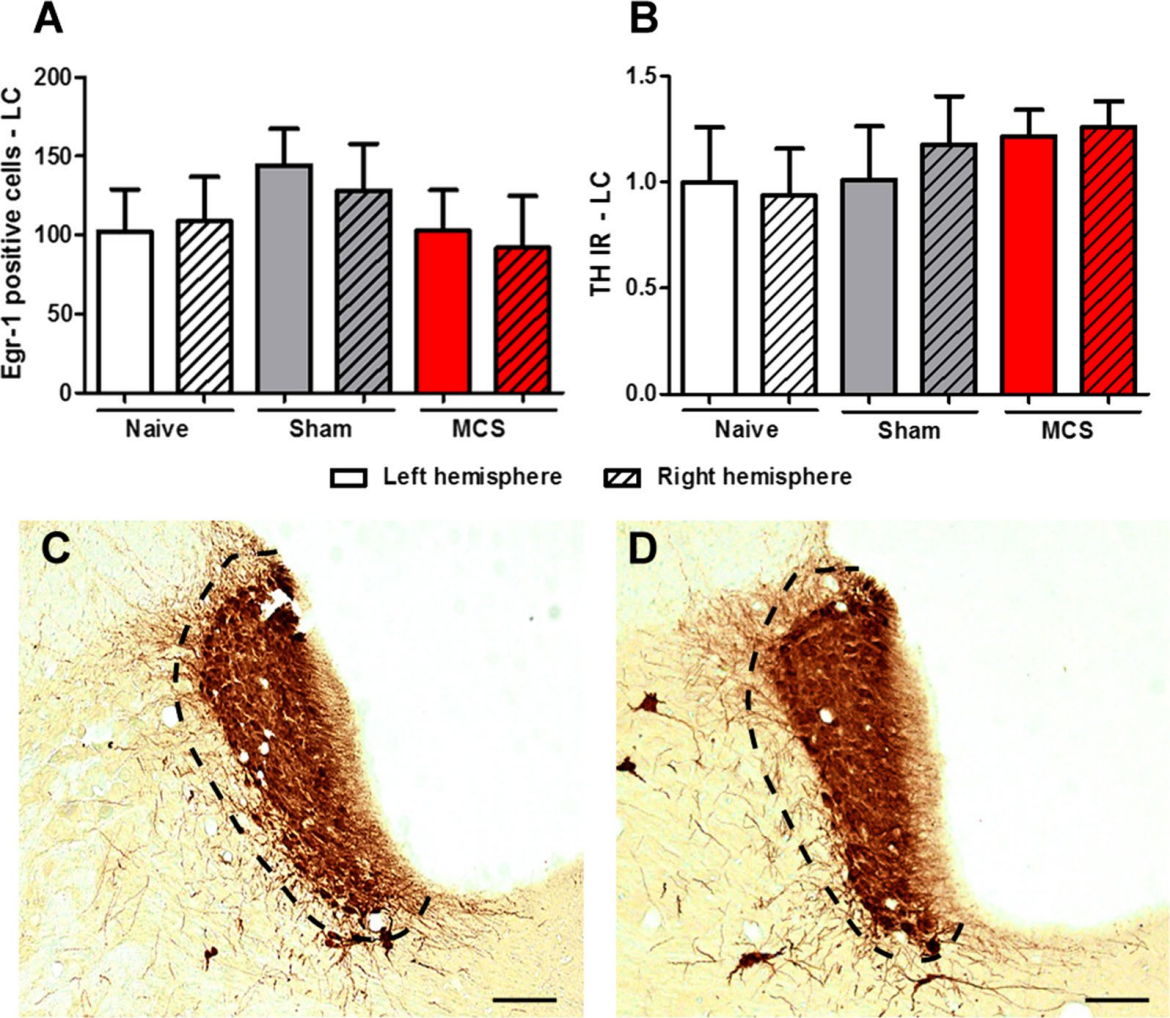
Involvement of LC in antinociception induced by MCS. Quantification of Egr-1 (**A**) and TH (**B**) IR in the LC of naive (without surgical intervention), sham (with epidural electrodes but without stimulation) and stimulated (MCS, 1.0 V/60 Hz/210 µs, 15 min) rats. Values represent the mean ± SEM (n = 5 animals per group). Photomicrographs illustrating the TH-IR in the LC of sham (**C**) and stimulated (**D**) rats. *IR* immunoreactivity, *LC* locus coeruleus, *MCS* motor cortex stimulation, *TH* tyrosine hydroxylase. Scale bars: 200 µm

In the DRN, we observed the same staining intensity for Egr-1 (Fig. 3A) and for 5HT (Fig. 3B) between naive animals and sham-stimulated animals. MCS did not interfere with DRN activity (1-w-ANOVA, F_(2,12)_ = 0.74, p = 0.4945; Fig. 3A); however, it induced an increase of 70% in the 5HT-IR of the DRN compared with that of the naive group (1-w-ANOVA, F_(2,12)_ = 8.42, p = 0.0029, followed by Bonferroni’s *post hoc* test, p < 0.01; Fig. 3B–D; Additional file 1: Table S1). Within the LC, animals submitted to MCS presented similar staining of Egr-1-positive neurons to naive and sham-stimulated animals in both cerebral hemispheres (1-w-ANOVA, F_(5,25)_ = 0.49, p = 0.7797; Fig. 4A) and TH-IR (1-w-ANOVA, F_(5,25)_ = 0.42, p = 0.8285; Fig. 4B–D; Additional file 1: Table S1).

**Fig. 5:**
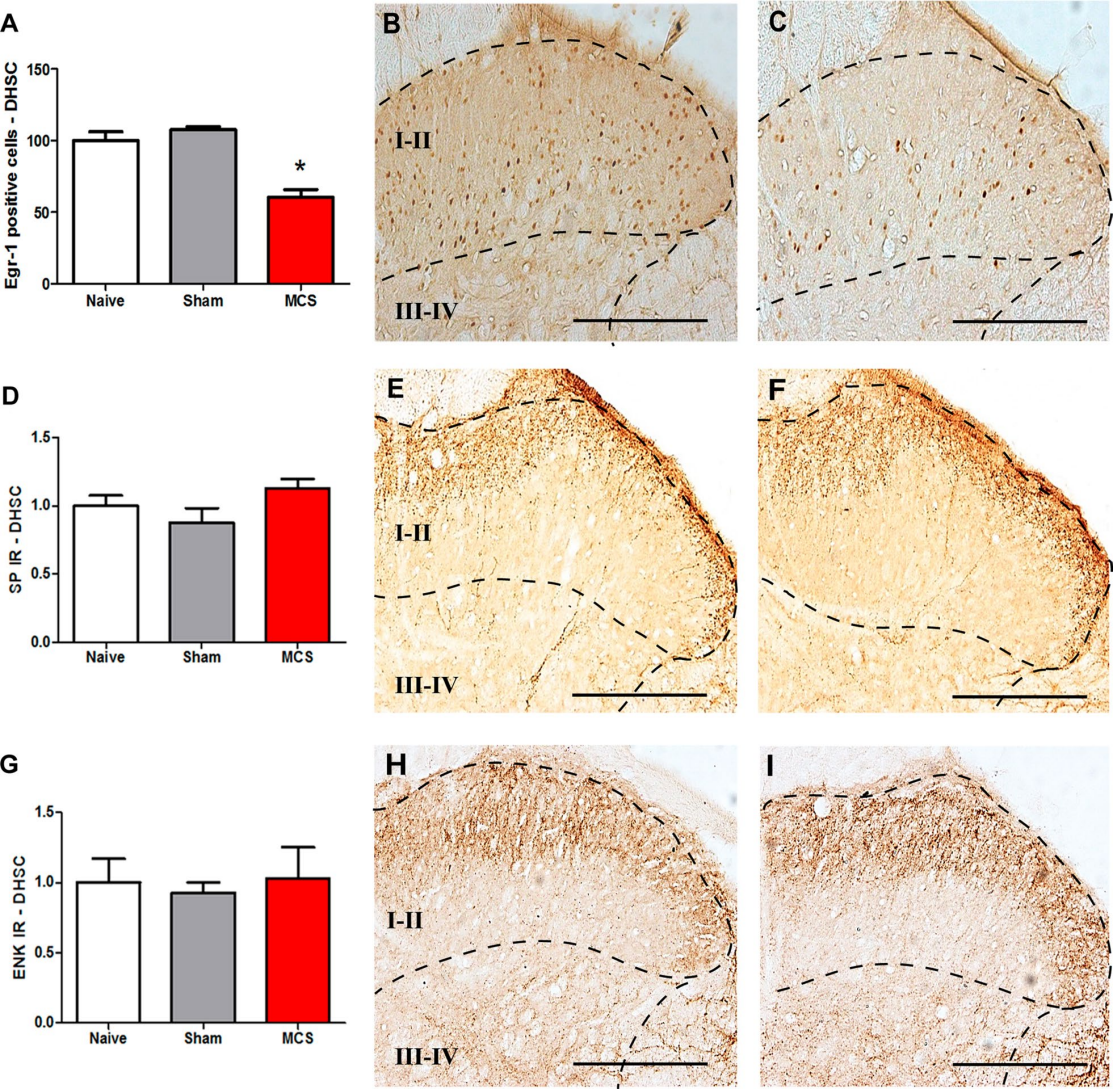
Effect of cortical stimulation on DHSC activation. Quantification of Egr-1 (**A**), SP (**D**) and ENK (**G**) IR in the DHSC of naive (without surgical intervention), sham (with epidural electrodes but without stimulation) and stimulated (MCS, 1.0 V/60 Hz/210 µs, 15 min) rats. Values represent the mean ± SEM (n = 5 animals per group). *p < 0.05 compared to the naive group, after Bonferroni’s *post hoc* test. Photomicrographs illustrating Egr-1-IR (**B**, **C**), SP-IR (**e**, **F**) and ENK-IR (**H**, **I**) in sections of the DHSC (laminae I-IV, right side) from sham (**B**, **E**, **H**) and stimulated (**C**, **F**, **I**) rats. *DHSC* dorsal horn of the spinal cord, *ENK* enkephalin, *IR* immunoreactivity, *MCS* motor cortex stimulation, *SP* substance P. Scale bars: 200 µm

Concerning the spinal cord (laminae I–IV), animals subjected to MCS showed a decrease of 72% in the Egr-1-IR in the DHSC compared to the level in the naive group (1-w-ANOVA, F_(2,12)_ = 26.90, p = 0.0002; followed by Bonferroni’s *post hoc* test, p < 0.05; Fig. 5A–C; Additional file 1: Table S1). Moreover, MCS did not change the pattern of SP-IR (1-w-ANOVA, F_(2,12)_ = 2.40, p = 0.2423; Fig. 5D–F) and ENK-IR (1-w-ANOVA, F_(2,12)_ = 0.0978, p = 0.9075; Fig. 5G–I) in the DHSC compared with the patterns in naive animals. Naive animals showed the same staining pattern for Egr-1, SP and ENK as sham-stimulated animals (Fig. 5A, D, G) (Additional file 1: Table S1).

## Discussion

### Are serotoninergic nuclei involved in MCS-induced antinociception in the absence of neuropathic conditions?

MCS alleviates neuropathic pain in humans and animals; nevertheless, the subcortical relay mechanisms involved in this response are not yet fully understood. In human studies, it has been suggested that the neurocircuitry involved in the emotional component of pain and in the descending analgesic system mediate analgesia induced by MCS [21, 39, 70, 71]. In naive and neuropathic rats, MCS-induced antinociception is accompanied by PAG activation [64, 65], which plays a pivotal role in the activation of descending analgesic pathways [55]. The antinociceptive response observed in naive rats is specific to motor cortex, considering that stimulation of posterior parietal or somatosensory cortices did not elicit any changes in the general activity or nociceptive response [12]. In neuropathic rats, MCS-induced analgesia is modulated by the action of RVM on spinal 5HT_1A_ receptors [100]; however, the role of motor cortex in the activation of the descending serotonergic nuclei, in healthy conscious rats, has not been evaluated yet.

Neuronal activity mapping with inducible transcription factors (ITFs) or immediate early genes, such as c-fos, c-jun and egr-1 (zif268, krox-24 or zenk), has been widely used to study the neurocircuitry underlying nociception [28–30, 35, 38]. Although this tool is widely used, it is limited in the interpretation of the cell type and mechanism of action; however, it makes it possible to determine which structures are more or less activated in relation to the applied intervention, allowing for a more detailed investigation in this altered area. ITFs showed to be overexpressed in neurons in response to extracellular stimuli including peripheral nociceptive stimulation in different areas of the brain and spinal cord [26, 28, 38, 83]. Supporting earlier findings [12, 16, 65, 78, 99], we showed here that MCS raised the mechanical nociceptive threshold of the hind paw contralateral to the stimulation side in healthy, conscious animals. To substantiate the hypothesis of the role of the descending serotoninergic system in MCS-induced antinociception, we investigated Egr-1-IR and 5HT-IR in the NRM, a critical relay for descending pain control [55]. The NRM contains serotonergic neurons, which exert facilitatory and inhibitory influence on spinal nociceptive transmission [11, 63, 79]. The NRM serotonergic neurons are considered essential for descending analgesic control, and although they constitute only approximately 20% of the total population of the NRM, they are one of the main sources of 5HT to the DHSC [9, 54]. Our results showed that, in healthy animals, MCS not only induced activation of the NRM, observed by Egr-1 labeling, but also increased the 5HT production within this nucleus. Considering that RVM blockade has spinal nociceptive effects in healthy control animals [22, 27], we can suggest that the NRM is activated after cortical stimulation and that this response may contribute to the elevation of the nociceptive threshold in healthy rats.

The DRN, the major source of serotonergic neurons in the brain [36, 92], is another important nucleus involved in pain control [18, 63, 101]. The DRN projections to the forebrain area contribute effectively to the affective-motivational component of pain [101], and since MCS-induced analgesia appears to specifically modulate the emotional appraisal of pain, rather that its intensity [70], we hypothesized that the DRN may also be involved in the effect of MCS. To test this hypothesis, we assessed Egr-1 and 5HT immunolabeling in the DRN after MCS-induced antinociception. We observed here that MCS increased the amount of 5HT staining in the DRN but did not interfere with the pattern of neuronal activation in this nucleus. The DRN has a high degree of neuronal heterogeneity; this nucleus comprises groups of neurons containing 5HT, glutamate, ENK, GABA and dopamine, which may be combined with different neuropeptides [19, 46, 84] and this neuronal diversity is not restricted to inhibition; some DRN neurons are involved in facilitation of nociceptive transmission [101]. The DRN synaptic circuits are finely regulated by glutamate and GABA arising from extra-raphe areas as well as from local sources, influencing the activity of 5HT cells [84]. Regarding nociceptive inhibition, serotonergic DRN neurons receive excitatory glutamatergic connections, leading to 5TH release directly to the DHSC or indirectly to the NRM, modulating the spinal nociceptive response [101]. The inhibition of nociceptive response depends on the activation of 5-HT receptor subtype and its anatomical location [34, 55]. In this sense, under healthy conditions, the activation of spinal 5HT_1A_ receptor inhibits the nociceptive response in the DHSC [23]. Concerning nociceptive facilitation, serotonergic DRN neurons receive inhibitory connections from GABAergic neurons, which contribute to the inhibition of 5HT release within this nucleus [84, 86, 101]. As no changes in neuronal activity were found in the DRN following MCS, it is plausible that the sum of the inhibitory and excitatory signals within the DRN amounts to zero, reflecting balanced neuronal changes that modulate the activity of this nucleus. In this regard, our hypothesis is that the MCS inhibits the local GABAergic interneurons, leading to an increase in the activity of 5HT cells, resulting in a lack of change in the pattern of neuronal activation in the DRN. This hypothesis is supported by the fact that the MCS-induced antinociception was accompanied by considerable enhancement of 5HT in the DRN, converging with other studies that showed an increase in 5HT or its synthesizing enzyme tryptophan hydroxylase in analgesic conditions [66, 87, 101, 104]. Our data suggest for the first time that the DRN, an important nucleus involved in the emotional appraisal of pain, can play a significant role in the elevation of the nociceptive threshold after MCS in healthy rats.

### Is the LC not really involved in descending analgesic control induced by MCS?

The brainstem nuclei A5, LC (A6) and A7 are the main sources of the noradrenergic nerve terminals in the spinal cord [37, 103], with the LC providing the predominant noradrenergic input to the DHSC [5, 33, 72]. The LC exerts a predominant inhibitory effect on spinal nociceptive transmission by descending noradrenergic fibers; however, it also exerts a facilitatory effect on the nociceptive response by ascending fibers [37, 43, 68, 69, 107]. Since the noradrenergic neurons of the LC are critical for descending analgesic control [37, 68], we evaluated the pattern of Egr-1-IR and TH-IR (for NA neurons) in this area. Our data showed that MCS did not change neuronal activation or TH staining in the LC compared with the values of the non-operated naive animals.

It was previously shown that MCS increased the discharge rates of LC neurons in neuropathic rats; however, it did not change the neuronal firing rates of LC cells in sham-operated animals [99]. Additionally, the same authors showed that local pharmacological inactivation of the LC and blockade of spinal α2-adrenergic receptors failed to reverse MCS-induced antinociception in the neuropathic and sham-operated rats, suggesting that the descending noradrenergic pathway originating in the LC may do not play a crucial role in spinal antinociception induced by cortical stimulation [99]. Under healthy conditions, the coeruleospinal noradrenergic system has only a slight influence on nociceptive response, whereas, with sustained pain and pathophysiological states, it plays a critical role in pain control [25, 58, 68, 94, 95]. On this topic, it was shown that blockade of α2-adrenergic receptors and lesion of the LC had no effect on spinal nociceptive neurons in healthy animals but modulated the magnitude and duration of the neuronal responses in animals with peripheral inflammation [25, 94]. Our results emphasize the idea that the coeruleospinal noradrenergic pathway might play a secondary role in the control of nociceptive response under basal conditions, particularly in MCS-induced antinociception.

### How is the spinal circuitry affected in response to elevation of the nociceptive threshold after MCS?

The DHSC is the complex site where several ascending and descending sensory pathways modulate nociceptive information, acting on projection neurons, primary afferent neurons and excitatory and inhibitory interneurons to contribute to pain processing in both facilitatory and inhibitory systems [89, 90, 105]. Considering that the activation of descending serotonergic and noradrenergic analgesic pathways results in inhibition of the firing of spinal nociceptive-specific neurons in healthy conditions [10, 41, 106] and that the involvement of these pathways in the MCS-induced antinociception is very clear in the literature and corroborate with our findings, we investigated the spinal modulation after MCS. For that, we applied Egr-1-IR to evaluate the pattern of neuronal activation in the DHSC after cortical stimulation. We showed here that MCS decreased the Egr-1-positive neurons in the DHSC, corroborating the idea that there is a direct correlation between a decrease in ITF expression and antinociception in the DHSC [7, 24, 57]. In line with this idea, we showed previously in neuropathic rats that the MCS-induced analgesia is accompanied by complete reversion of spinal hyperactivity induced by peripheral neuropathy, which was manifested by a decrease in Egr-1-IR in the spinal cord [64]. Moreover, MCS in healthy rats attenuated the neuronal discharge rates [75, 80] and Egr-1-IR [16] in the DHSC in response to peripheral mechanical stimulation. Our results support the hypothesis that MCS inhibits the DHSC neurons directly through activation of corticospinal pathways and/or indirectly through activation of the descending analgesic pathways.

SP is a neuropeptide that binds to neurokinin 1 (NK1) receptors and plays an important role in the transmission of nociceptive signals from primary afferent neurons in the spinal cord, contributing to excitability of projection neurons and central sensitization [2, 3, 51, 82]. In the spinal cord, SP is present in interneurons, descending fibers, and central terminals of the primary afferent neurons located superficially in the DHSC [31, 77, 81]. Taking into account that SP is one of the main excitatory neurotransmitters released in the DHSC that mediate nociceptive transmission from the peripheral to the central nervous system, we evaluated the pattern of immunolabeling for SP in the DHSC in response to MCS. In the DHSC, 80% of SP is in primary afferent terminals and is co-localized with NK1 in projection neurons in lamina I of spinal cord [31, 52, 91]. Corroborating these findings, we also observed a high concentration of SP in superficial laminas of the DHSC; however, no difference was observed in the staining intensity of this neuropeptide among naive, sham-stimulated and stimulated animals.

The intensity and modality of a stimulus and the degree of inflammation it induces are critical issues that determine spinal SP release and the extent to which this neuropeptide contributes to the transmission of nociceptive signals [1, 40, 49]. Occupancy of NK1 leads to internalization of the receptor, which has been used as a quantitative indicator of SP release by sensory neurons in response to noxious stimuli [1, 49, 50, 53]. However, it was identified by tracking NK1 internalization in the DHSC neurons that SP is released only under conditions of intense pain [2]. In addition, the loss of NK1-expressing spinal neurons decreases the nociceptive hypersensitivity associated with chronic pain conditions; however, responses to nociceptive stimuli of lower intensity are unaffected by this loss [61], suggesting that the SP-NK1 system is not crucial to spinal nociceptive transmission under healthy conditions. Considering our findings that MCS inhibits the extent of spinal neuronal activation and does not alter SP immunoreactivity in the DHSC, we can hypothesize the following: (1) The modulation of SP action can occur postsynaptically, with direct inhibition of NK1 receptor internalization within projection neurons; (2) Under basal conditions, in the absence of intense painful stimuli, the inhibition of projection neurons appears not to be related to the inhibition of SP release from primary afferent fiber terminals, and the inhibition of other neurotransmitters such as glutamate or CGRP may be the main drivers of this antinociceptive effect; (3) The control of spinal nociceptive transmission in the absence of persistent pain can rely more on the activation of inhibitory systems in the spinal cord circuitry than on the inhibition of excitatory systems.

A brainstem-spinal cord inhibitory circuit controls the mechanical pain threshold through ENK- and GABA-mediated inhibition of spinal nociceptive neurons [17]. Taking into account the crucial role of ENK in the inhibition of the spinal projection neurons [6, 76] and the involvement of the opioid system in MCS-induced antinociception in healthy rats [12], we investigated ENK labeling in the spinal cord in response to cortical stimulation. Our results are consistent with a previous report demonstrating that in the spinal cord, the highest concentrations of ENK-positive fibers are observed in laminae I and II [32]; therefore, we did not detect any changes in ENK labeling between the different experimental groups. There are several polysynaptic inhibitory circuits in the DHSC that can be critical in controlling the spinal nociceptive neurons. Further investigation of the spinal neurocircuitry is required to understand how the motor cortex modulates the nociceptive threshold in the spinal cord in the absence of persistent pain.

## Conclusion

Taken together, our results suggest that MCS induces the activation of serotonergic NRM and DRN as well as the inhibition of spinal neurons, and such effects may contribute to the elevation of the nociceptive threshold in healthy conscious rats. Moreover, MCS-induced antinociception, under baseline conditions, may not involve both the noradrenergic coeruleospinal pathway and changes in SP or ENK release in the spinal cord. A better understanding of how these areas respond to MCS under normal conditions will help clarify the role of motor cortex in pain control and how malfunctions of that region could drive responsiveness to cortical stimulation under neuropathic conditions. Our findings expand the scientific knowledge regarding the role of primary motor cortex in pain control, emphasizing that it may be one of the most rostral structures in the neuroaxis related to the pain modulatory system.

## Additional file

**Additional file 1: Table S1.** Pain behavior and immunoreactivity data from naive, sham and stimulated rats.

## Abbreviations

5HT: serotonin
ACC: anterior cingulate cortex
DHSC: dorsal horn of the spinal cord
DRN: dorsal raphe nucleus
ENK: enkephalin
IR: immunoreactivity
LC: locus coeruleus
MCS: motor cortex stimulation
NA: noradrenaline
NRM: nucleus raphe magnus
PAG: periaqueductal gray
RVM: rostral ventromedial medulla
SP: substance P
TH: tyrosine hydroxylase
