# Supplementary material for: Motor cortex and pain control: exploring the descending relay analgesic pathways and spinal nociceptive neurons in healthy conscious rats
Source: Behav Brain Funct. 2019 Mar 25;15:5. doi: 10.1186/s12993-019-0156-0 (PMC6432755; doi:10.1186/s12993-019-0156-0)
Supplement: Supplementary file 1 — Additional file 1: Table S1. Pain behavior and immunoreactivity data from naive, sham and stimulated rats. [file 12993_2019_156_MOESM1_ESM.docx]

**Table S1.**

Pain behavior and immunoreactivity data from naive, sham and stimulated rats.

|  | **Naive** | | **Sham** | **MCS** | **F** | **p value** |
| --- | --- | --- | --- | --- | --- | --- |
| **Nociceptive threshold** | |  |  |  |  |  |
| IM | 65 ± 2.67 | | 68 ± 2.98 | 66 ± 3.05 | - | - |
| FM1 | 61 ± 1.75 | | 59 ± 3.20 | 59 ± 2.00 | - | - |
| FM2 | 59 ± 1.83 | | 53 ± 4.00 | 96 ± 3.75 | 24.91 | < 0.0001* |
| **DRN** |  | |  |  |  |  |
| Egr-1 positive cells | 100 ± 11.02 | | 79 ± 7.63 | 101 ± 18.00 | 0.7361 | 0.4945 |
| 5HT-IR | 1.00 ± 0.04 | | 1.08 ± 0.09 | 1.79 ± 0.19 | 8.422 | 0.0029* |
| **NRM** |  | |  |  |  |  |
| Egr-1 positive cells | 100 ± 11.90 | | 109 ± 5.91 | 170 ± 8.86 | 20.06 | 0.0003* |
| 5HT-IR | 1.00 ± 0.16 | | 0.85 ± 0.08 | 1.61 ± 0.18 | 6.432 | 0.0126* |
| **LC** |  | |  |  |  |  |
| Egr-1 positive cells | 100 ± 26.66 | | 144 ± 22.95 | 103 ± 25.59 | 0.4896 | 0.7797 |
| TH-IR | 1.00 ± 0.26 | | 0.91 ± 0.25 | 1.11 ± 0.13 | 0.4192 | 0.8285 |
| **DHSC** |  | |  |  |  |  |
| Egr-1 positive cells | 100 ± 6.17 | | 108 ± 2.02 | 60 ± 5.45 | 26.90 | 0.0002* |
| SP-IR | 1.00 ± 0.07 | | 0.88 ± 0.11 | 1.12 ± 0.07 | 2.398 | 0.2423 |
| ENK-IR | 1.00 ± 0.17 | | 0.93 ± 0.08 | 1.03 ± 0.23 | 0.09782 | 0.9075 |

Values represent the means ± SEM (n = 10 animals/group for nociceptive threshold analysis and n = 5 animals/group for immunohistochemistry analysis).

*p < 0.05 compared to the naive group.
